# A rare case of unicentric Castleman disease in the posterior mediastinum: case report and integrated literature review

**DOI:** 10.3389/fonc.2026.1716642

**Published:** 2026-03-24

**Authors:** Lirong Zhang, Shaomeng Yang, Lan Fang, Huaiyu Zhang

**Affiliations:** 1 Department of Radiology, Aerospace Center Hospital, Beijing, China; 2 Department of Pathology, Aerospace Center Hospital, Beijing, China; 3 Department of Radiology, PLA Rocket Force Characteristic Medical Center, Beijing, China

**Keywords:** Castleman disease, diagnostic imaging, posterior mediastinum, surgical resection, unicentric

## Abstract

**Background:**

Castleman disease (CD) is a complex lymphoproliferative disorder characterized by nonspecific clinical manifestations and imaging features. When occurring within the mediastinum, it is easily confused with other diseases, posing diagnostic challenges. We report a case of unicentric hyaline vascular Castleman disease (UCD) located in the posterior mediastinum. This case report is supplemented by a retrospective analysis of the literature, aiming to enhance understanding and improve diagnostic accuracy for this condition.

**Case summary:**

This paper provides a detailed account of the clinical data, imaging findings, diagnostic and therapeutic process of a 28-year-old female patient with a posterior mediastinal UCD. Seven years ago, the patient incidentally found a left posterior mediastinal mass during an outpatient visit for a respiratory tract infection at another hospital. A needle biopsy at that time suggested post-infectious reactive lymphadenopathy. After antimicrobial therapy, respiratory symptoms improved, but intermittent back pain subsequently occurred without prompt attention and follow-up. The patient recently presented for follow-up due to persistent left-sided chest pain unresponsive to analgesics. Imaging examination revealed a significantly enlarged lesion compared to previous findings, now extending to the left thoracic ninth intervertebral foramen region. Additionally, localized increased bone density was observed adjacent to the ninth rib. The patient ultimately underwent complete surgical resection, with postoperative pathology confirming hyaline vascular Castleman disease. No adjuvant radiotherapy or chemotherapy was administered postoperatively.

**Conclusion:**

The definitive diagnosis of posterior mediastinal hyaline vascular type CD relies on histopathological examination of surgically resected specimens combined with immunohistochemical staining to establish diagnosis and classification. Imaging features of a hyper-vascular mass in the posterior mediastinum, exhibiting persistent marked enhancement with peri- and intra-tumoral tortuous feeding vessels, may provide valuable diagnostic clues for this disease. Radiologists and relevant clinicians should enhance the recognition of this entity to facilitate the formulation and optimization of diagnostic and therapeutic strategies.

## Introduction

Castleman disease (CD) is a complex lymphoproliferative disorder, also known as angio-follicular lymphadenopathy (1), First described by Benjamin Castleman in 1954. The disease can be classified into two main clinical types: unicentric Castleman disease (UCD) and multicentric Castleman disease (MCD) (2). The most common site of involvement is the thorax (30%–70%)​, followed by the neck (10%–40%), the abdomen and pelvis (12%–39%), and the axilla (4%–5%). Other sites are less frequently involved (2). Approximately 70% of UCD cases occur within the thoracic cavity, with the majority located in the mediastinum (3). UCD is a lymphoproliferative disorder that can arise in any mediastinal compartment containing lymphoid tissue. It most frequently occurs in the anterior and middle mediastinum, while origination from the posterior mediastinum is considerably rare (4). We present a case of UCD primarily located in the posterior mediastinum, which clinically and radiologically mimicked a neurogenic tumor, thereby posing significant challenges for preoperative diagnosis. Through a literature review, we aim to enhance understanding of its clinical characteristics and diagnostic strategies.

## Case report

### Case description

**Figure 1:**
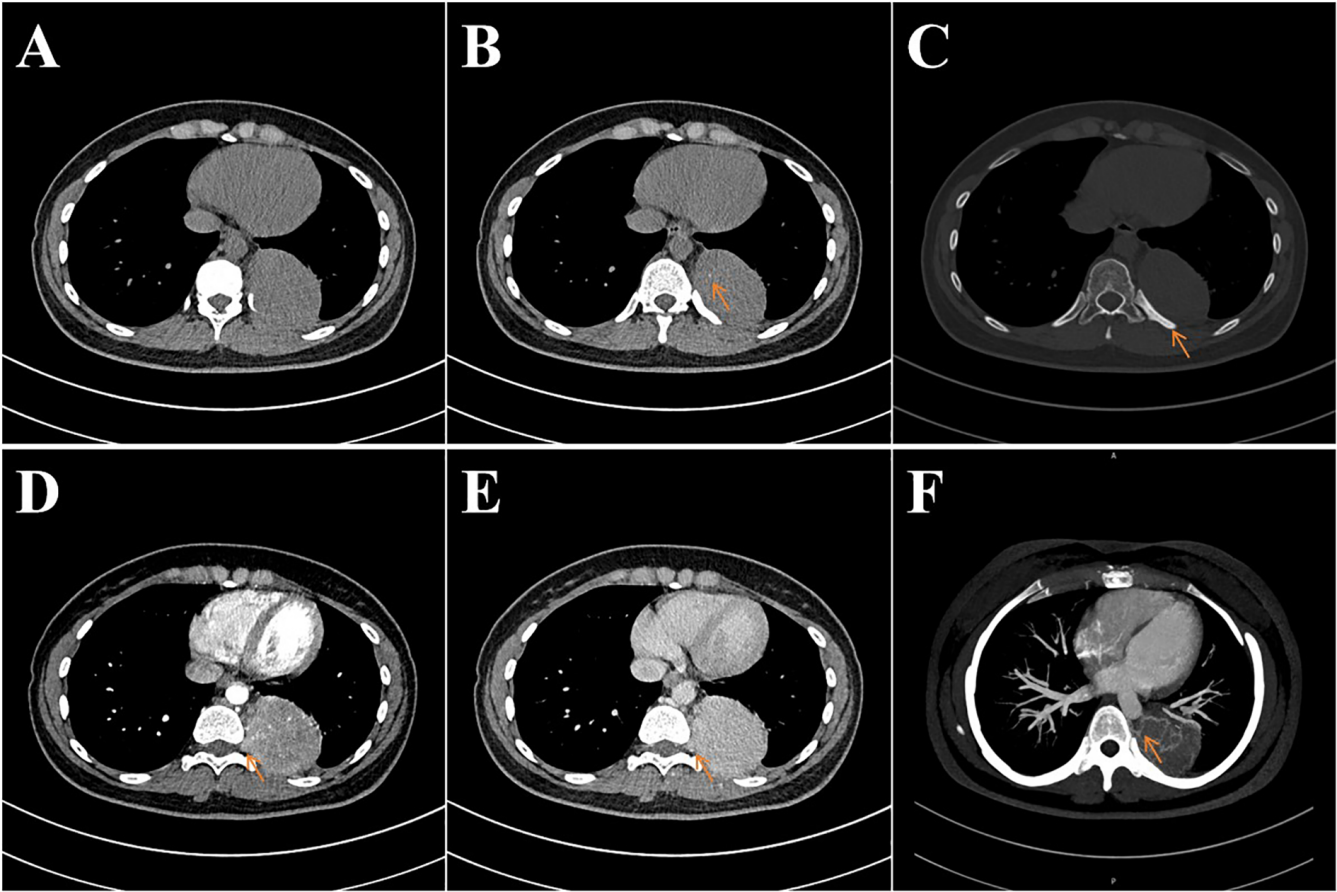
The patient’s plain and contrast-enhanced CT images. **(A)** shows a well-defined soft tissue density mass at the left posterior mediastinal border. **(B)** demonstrates fine punctate calcifications within the lesion. **(C)** reveals increased bone density in the adjacent ribs. **(D, F)** indicate involvement of the left T9 intervertebral foramen region, with persistent marked enhancement after contrast administration. **(F)** demonstrates thickened and tortuous feeding arteries (originating from the thoracic aorta) both peritumorally and intratumorally.

The patient was a 28-year-old Asian female with no family history of lymphoproliferative disorders, who was incidentally found to have a left posterior mediastinal mass during an outpatient visit seven years ago for a respiratory tract infection. At that time, a needle biopsy suggested “post-infectious reactive lymphadenopathy.” After antimicrobial therapy, her respiratory symptoms improved, but she subsequently experienced intermittent back pain that was not taken seriously, and no follow-up examinations were conducted during this period due to the mild and transient nature of the symptoms. The patient recently presented for follow-up due to persistent left-sided chest pain. Physical examination revealed tenderness over the left thoracic spine without neurological deficits. The patient reported ineffective pain relief from analgesics. Imaging examination revealed a well-defined left posterior mediastinal mass lesion at the T9-T11 vertebral level, measuring approximately 5.8 × 5.9 × 6.2 cm. The lesion caused enlargement of the left T9 intervertebral foramen. The lesion demonstrated uniform density with no evidence of necrosis or cystic changes. Minor calcifications were noted within the lesion. Following contrast enhancement, the lesion demonstrated persistent, marked enhancement with arterial phase computed tomography(CT) values of approximately 100-110 Hounsfield Units (HU) and venous phase CT values of approximately 130-145 HU. Thickened and tortuous vascular shadows were visible both within and surrounding the lesion. Increased bone density was observed in the adjacent 9th rib (**Figure 1**).

## Therapeutic intervention

The patient underwent resection of a mediastinal mass under general anesthesia via video-assisted thoracoscopic surgery (VATS). Positioned in the right lateral decubitus position, after achieving adequate anesthesia, a 2-cm skin incision was made at the 5th intercostal space along the anterior axillary line on the left side. Subcutaneous tissue and muscles were sequentially dissected, and a thoracoscope was inserted. The tumor was located superior to the diaphragm, paraspinal, with a broad base and rich vascular supply, suggesting difficulty in endoscopic resection. A 5-cm skin incision was made along the left posterior axillary line at the level of the eighth rib. The subcutaneous tissue and muscular layers were sequentially divided to access the thoracic cavity. Upon exploration, the tumor was found to have invaded the adjacent rib. The ninth rib was meticulously mobilized, and a partial resection of the ninth rib was performed to facilitate exposure. The tumor capsule was then incised, revealing significant hemorrhage upon manipulation. Due to the tumor’s broad base, rich vascularity, and invasion of the ninth rib, conversion to open thoracotomy was deemed necessary. Intraoperative blood loss totaled 1000 mL, managed with transfusion of two units of packed red blood cells. A partial excision of the tumor tissue was initially performed, and the specimen was sent for intraoperative frozen section analysis. Following pathological confirmation of unicentric Castleman disease (hyaline vascular variant), the remaining tumor tissue was completely resected. The thoracic cavity was thoroughly irrigated, and a 24-French chest tube was placed through the port site for postoperative drainage. Hemostasis was ensured, and the wound was closed in layers.

## Pathological findings and immunohistochemistry

**Figure 2:**
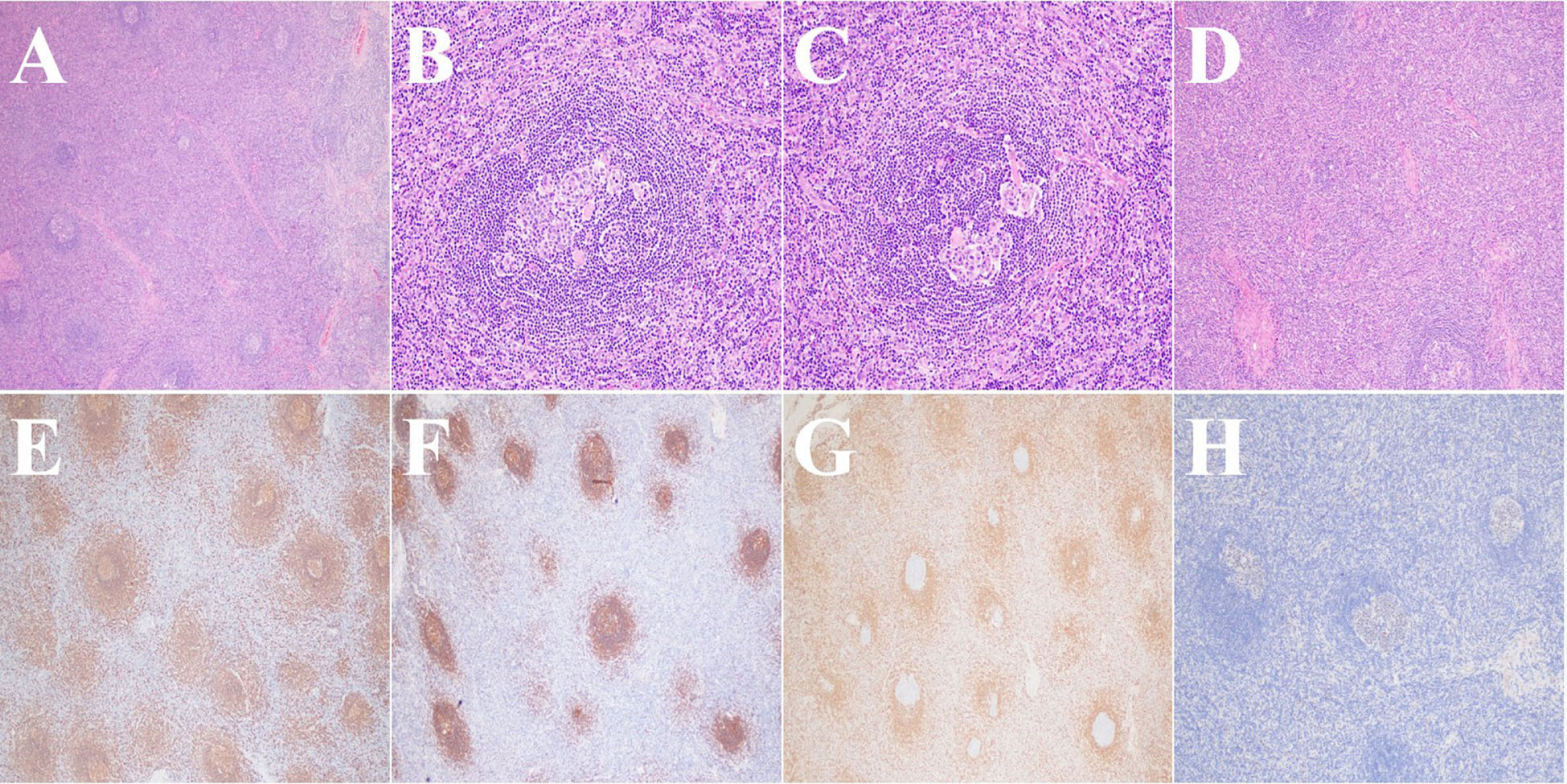
Histopathological and immunohistochemical features. **(A)** shows atrophic germinal centers (GC); **(B)** demonstrates concentric lymphocyte arrangement around the GC with an “onion-skin” appearance; **(C)** shows hyalinized vessels penetrating the GC; **(D)** shows vascular proliferation in the interfollicular areas with thickened walls and hyalinization; **(E)** shows CD20 staining positivity; **(F)** shows CD21 staining positivity; **(G)** shows Bcl-2 positivity outside the follicles; **(H)** shows Bcl-6 staining positivity.

Intraoperative and postoperative (mediastinal mass) pathology revealed proliferative lymphoid lesions with partial atrophy of follicular germinal centers and numerous hyalinized vessels. Immunohistochemistry results: CD3 (T-cell positive), CD20 (B-cell positive), CD21 (Follicular dendritic cell, FDC, positive), Bc1-2 (extrafollicular positive), Bcl-6 (germinal center, GC, positive), CD10 (GC positive), CD5 (T-cell positive), Ki-67 (40% positive), CD23 (FDC positive), CD138 (focal positive), CD34 (vascular positive); *in situ* hybridization: Epstein-Barr virus-encoded small RNA (EBER) (-). Based on morphology and immunohistochemistry, the diagnosis was Castleman disease, hyaline vascular variant (**Figure 2**). The Ki-67 proliferation index was approximately 40%, predominantly exhibiting focal distribution and confined to follicular germinal centers. This pattern characteristically reflects active lymphoproliferation in clear vascular monocentric disease and should not be interpreted as a malignant indicator. Furthermore, the EBER-negative result, combined with the clinical presentation of a solitary mass and classic histopathological features, strongly supports the diagnosis of Human Herpesvirus 8 (HHV-8)-negative monocentric disease. Consequently, HHV-8 testing was not performed.

## Postoperative follow-up

The patient’s postoperative course was uneventful. She was regularly followed up for a period of 6 months with scheduled clinical evaluations and imaging examination. Throughout this period, the patient reported complete resolution of her preoperative symptoms and showed no signs of recurrence on surveillance imaging. For UCD treated with complete resection, clinical and imaging follow-up was suggested every 6-12 months for the first 2-3 years. The patient will continue long-term monitoring according to this protocol.

## Discussion

We report the clinical and imaging data of a young female patient with UCD located in the posterior mediastinum. The clinical history spanned seven years, characterized by an initial absence of significant symptoms followed by progression to persistent chest pain that was refractory to analgesic medication. Imaging studies revealed a hyper-vascular space-occupying lesion in the posterior mediastinum. Its growth pattern mimicked that of a neurogenic tumor and was associated with enlargement of the left T9 intervertebral foramen.​​ Morphologically, the lesion demonstrated a ​vertical-to-transverse diameter ratio greater than 1. ​Contrast-enhanced imaging​ revealed ​persistent and marked enhancement. ​Prominent, tortuous feeding vessels​ were observed ​both within the lesion and in its periphery. The initial misdiagnosis from the needle biopsy (core needle biopsy) as reactive lymphadenopathy highlights a key diagnostic pitfall. The histological diagnosis of hyaline-vascular UCD relies critically on the preservation of intact nodal architecture, including characteristic features like regressed germinal centers, concentric “onion-skin” lymphocyte layering, and interfollicular vascular proliferation. These architectural clues are often lost or inadequately represented in limited biopsy specimens. Therefore, even a core needle biopsy may be non-diagnostic, underscoring the necessity of complete surgical excision for definitive diagnosis (5). In conclusion, this patient presented with insidious onset, and the clinical symptoms and imaging findings lacked specificity, posing challenges for clinical diagnosis.

UCD primarily manifests as benign, localized lymphoid hyperplasia. When originating in the mediastinum, it is usually located in the anterior and middle mediastinum. Occurrence in the posterior mediastinum is rare, with most cases reported as individual case studies. A systematic literature search was conducted using PubMed, Embase, and Web of Science databases. The search scope covered publications up to September 2025. Search terms included “posterior mediastinal Castleman disease,” “unicentric Castleman disease,” and “mediastinum.” Inclusion criteria were case reports or series describing unicentric CD localized to the posterior mediastinum. Eighteen cases meeting these criteria were identified and their clinical, imaging, and pathological features were summarized (**Table 1**). The age of onset ranged from 10 to 72 years, with a higher prevalence among females (11/18). Clinical manifestations were non-specific, ranging from asymptomatic incidental discoveries to localized compressive symptoms in symptomatic cases, including chest or back pain (4/18) and respiratory symptoms (3/18). Rare presentations included autoimmune-related manifestations such as myasthenia gravis and pseudotumor cerebri. Imaging studies revealed calcifications in 4/18 cases. The predominant enhancement patterns were marked or homogeneous enhancement (8/18). Prominent tortuous feeding vessels surrounding the lesion were reported in 5/18 cases. Aggressive growth with intervertebral foramen invasion was observed in 4 cases, mimicking neurogenic tumors. Necrosis or cystic changes were uncommon (2/18).

**Table 1:** Summary of clinical characteristics and outcomes in 18 cases of posterior mediastinal unicentric Castleman disease.

Reference No.	Publication year	Authors	Age/sex	Location	Size	Calcification	Enhancement pattern	Foramen involvement	Treatment	Outcomes
1	2025	Belayachi B, et al (6)	58/F	Right posterior mediastinum	3.4 cm × 2.6 cm	NR	Marked	NA	Thoracotomy	No recurrence
2	2021	Chinthareddy RR, et al (7)	19/M	Left upper posterior mediastinum	12.0 cm ×10.0 cm	NR	Heterogeneous	NA	Thoracotomy	No recurrence
3	2019	Gupta V, et al (8)	28/F	Left paravertebral posterior mediastinum	5.8 cm × 4.9 cm	NR	Heterogeneous	Yes	Thoracotomy	NR
4	2018	Fein AS, et al (9)	21/F	Left posterior mediastinum	5.9 cm × 4.7cm	NR	Not reported	NA	Thoracotomy	No recurrence
5	2014	Lee BE, et al (10)	63/M	Right posterior mediastinum	13.4 cm × 9.5 cm	NR	Heterogeneous	NA	Right pneumonectomy	NR
6	2014	Bellolio JE, et al (11)	31/F	Posterior mediastinum	5.0 cm × 4.5 cm	NR	Homogeneous	Yes	Thoracotomy	No recurrence
7	2013	Cai J, et al (12)	28/M	Posterior mediastinum	9.2 cm × 6.0 cm	Yes	NR	Yes	Thoracotomy	No recurrence
8	2013	Alavi A, et al (13)	28/F	Posterior mediastinum	7.5 cm × 5.0 cm	NA	Marked	NA	Thoracotomy	No recurrence
9	2011	Shetty S, et al (14)	54/M	Between azygos vein and esophagus	5.0 cm × 2.5 cm	NA	Marked	NA	VATS	No recurrence
10	2011	Paci M, et al (15)	15/F	Left paravertebral posterior mediastinum	3.3 cm × 2.0 cm	NA	Homogeneous	Yes	Thoracotomy	No recurrence
11	2011	Michaelides M, et al (16)	41/M	Left costovertebral groove	7.5 cm × 4.5 cm	NA	Central nodular enhancement	NA	Thoracotomy	No recurrence
12	2010	Westphal FL, et al (17)	72/F	Left costovertebral groove	NR	Yes	NR	NA	Thoracotomy	No recurrence
13	2005	Nakajima D, et al (18)	42/M	Posterior mediastinum	4.5 cm × 3.0 cm	Yes	Heterogeneous	Yes	Thoracotomy	No recurrence
14	2010	Shah S, et al (19)	30/F	Left paravertebral posterior mediastinum	10.0 cm × 7.0 cm	NR	Homogeneous	NA	Thoracotomy	No recurrence
15	2001	Pereira TC, et al (20)	38/F	Paravertebral region	11.0 cm × 10.0 cm	Yes	Not reported	NA	Thoracotomy	No recurrence
16	2009	EA S, et al (21)	31/F	Left posterior mediastinum	5.7 cm ×4.3 cm	NR	Homogeneous	Yes	Thoracotomy	No recurrence
17	2007	MA F, et al (22)	19/F	Right paraspinal region	6.8 cm × 6.2 cm	NR	Heterogeneous	Yes	Thoracotomy	No recurrence
18	1996	G A, et al (23)	10/M	Left anterolateral epidural space at C6-T2 level	6.6 cm × 4.5 cm	NR	Moderate homogeneous	NA	Thoracotomy	No recurrence

VATS, Video-assisted thoracoscopic surgery;NR, Not reported in the original literature;NA, Not applicable. Enhancement pattern was categorized as “Homogeneous”, “Heterogeneous” or “Marked” based on descriptions in the original articles. The outcomes indicate the status at the last follow-up as reported. Tumor size is presented as the two largest dimensions reported in the original studies. For cases where three dimensions were provided, the two largest values are listed to standardize data presentation and facilitate comparison.

The gold standard for diagnosing UCD relies on histopathological examination of surgically resected specimens, combined with immunohistochemical staining for definitive diagnosis and classification (24), fine-needle aspiration biopsy, however, yields limited tissue samples, making it difficult to visualize characteristic structures such as intact lymphoid follicles or vascular proliferation patterns. This increases the risk of misdiagnosis, potentially leading to the mistaken interpretation of reactive lymph node hyperplasia (25). Imaging examination provides significant value in identifying and localizing lesions, but its radiographic features can easily be confused with common retrosternal tumors such as neurogenic tumors. Particularly in rare cases, UCD may mimic the growth pattern of neurogenic tumors, even invading and enlarging the neural foramen, posing challenges for preoperative differentiation. Both entities can occur in overlapping anatomical locations (8), and both may demonstrate heterogeneous enhancement on post-contrast imaging (26). However, UCD typically exhibits relatively homogeneous density with infrequent cystic change or necrosis, and often shows progressive marked enhancement. In contrast, neurogenic tumors usually demonstrate mild to moderate and heterogeneous enhancement. Notably, the presence of prominent, tortuous feeding vessels surrounding the mass, as observed in this case, is extremely uncommon in neurogenic tumors. This imaging finding provides a crucial diagnostic clue for UCD. Furthermore, UCD must be differentiated from paraganglioma, lymphoma, and solitary fibrous tumor. Although paraganglioma is also a hyper-vascular lesion, its characteristic ​​”salt and pepper” sign​ on MRI and clinical symptoms related to ​excessive catecholamine secretion​ (such as paroxysmal hypertension, palpitations, and headache) aid in its distinction (25). Lymphomas typically demonstrate mild to moderate enhancement and calcification is uncommon (27), It is different from the UCD enhancement intensity and the minute calcifications within the lesion in this case. Solitary fibromas may also exhibit marked enhancement, but this enhancement typically presents as a map-like heterogeneous pattern with visible low-density areas within the lesion. The lesions are often connected to the pleura via a broad base and may display a pseudo-capsule (28).

Complete surgical resection is the preferred treatment strategy for UCD, generally yielding favorable outcomes. For cases of UCD that are unresectable or pose high surgical risks, treatment regimens become more complex and may include corticosteroids, immunosuppressants, and targeted therapies such as anti-IL-6 receptor antibodies like certuximab or siltuximab (29), rituximab or chemotherapy may also be used when necessary (30). The intraoperative blood loss of approximately 1000 mL in this case highlighted the highly vascular nature of hyaline vascular UCD. Given that preoperative imaging primarily suggested a neurogenic tumor, and considering the inherent risk of catastrophic spinal cord ischemia due to non-target embolization into spinal arteries (e.g., the artery of Adamkiewicz), compounded by limited institutional experience with embolization and the need for urgent surgery due to the patient’s intractable severe chest pain refractory to medical management, the multidisciplinary team collectively decided to proceed with surgical resection without preoperative embolization. Complete tumor excision was successfully achieved with adequate blood product support. Postoperatively, back pain symptoms resolved completely. Only symptomatic treatments such as anti-inflammatory therapy were administered; no chemotherapy or immunotherapy was performed. For hypervascular tumors, preoperative arterial embolization is a well-established strategy to reduce intraoperative bleeding and facilitate safer (31), more complete resection (32).

Based on this case experience, we recommend that for cases with high clinical and radiological suspicion of posterior mediastinal UCD, particularly those with prominent feeding vessels, preoperative imaging (e.g., CT angiography) should be performed to delineate vascular anatomy. This should be followed by multidisciplinary discussion to evaluate the feasibility of preoperative embolization for selected cases, thereby potentially reducing intraoperative bleeding risks and enhancing overall procedural safety.

This study has several limitations that should be acknowledged. First, the retrospective design and single-institutional data source introduce inherent selection bias, potentially limiting the generalizability of our findings. While unicentric UCD typically follows an indolent course, the 6-month follow-up period precludes assessment of late recurrence risks. Second, immunohistochemical confirmation for HHV-8 was not performed; while pathological features are overwhelmingly consistent with classic HHV-8-negative hyaline-vascular UCD, the absence of this test remains a technical limitation. Thirdly, despite the posterior mediastinal location of the lesion, MRI was not obtained, which limited the comprehensive evaluation of neural foraminal involvement and soft-tissue characterization.

## Conclusion

In conclusion, we report a rare case of UCD originating from the posterior mediastinum, presenting both clinical and imaging findings. Through a systematic review of the literature, we found that posterior mediastinal UCD exhibits diverse clinical manifestations, ranging from asymptomatic cases to localized compression symptoms and even autoimmune symptoms. For similar cases, precise preoperative imaging evaluation and differential diagnosis are crucial. Although posterior mediastinal neurogenic tumors, lymphomas, paragangliomas, and solitary fibrous tumors may exhibit partial imaging similarities to UCD, the enhancement pattern of UCD and the presence of prominent, tortuous peri-tumoral and intra-tumoral vessels provide significant diagnostic clues. Surgical resection, as the primary treatment for UCD, has demonstrated its efficacy in this case. However, for complex or high-risk cases, multidisciplinary collaboration remains essential for developing treatment plans. Further accumulation of clinical data is needed to optimize diagnostic and therapeutic strategies.

## Data Availability

The original contributions presented in the study are included in the article/supplementary material. Further inquiries can be directed to the corresponding author.
